# Genome-Wide Identification of the *AMT* Gene Family in Wheat: Expression Profiles Under Ammonium Nutrition and Pathogen Effects

**DOI:** 10.3390/genes16121451

**Published:** 2025-12-04

**Authors:** Yanzhen Wang, Jialu Li, Xia Liu, Rui Huang, Menglin Lei, Yaoyuan Zhang, Guoqing Cui

**Affiliations:** 1 Center for Agricultural Genetic Resources Research, Shanxi Agricultural University, Taiyuan 030031, China; wangyanzhen9605@163.com (Y.W.); 19246278166@163.com (J.L.); liuxia1214lx@163.com (X.L.); huangrui@sxau.edu.cn (R.H.); leimenglin35@163.com (M.L.); 2 Shanxi Institute of Organic Dryland Farming, Shanxi Agricultural University, Taiyuan 030031, China

**Keywords:** wheat, ammonium nitrogen, *Puccinia striiformis*, *Blumeria graminis*

## Abstract

**Background**: Ammonium nitrogen (NH_4_^+^) serves as a vital nitrogen source, playing pivotal regulatory roles in plant growth, development, and high-yield formation. Ammonium transporters (AMTs), encoded by the *AMT* gene family, are central to NH_4_^+^ transport. However, the functional roles of *AMT* genes in wheat remain poorly understood. **Methods**: A comprehensive genome-wide analysis of the Ta*AMT* gene family numbers was conducted, encompassing investigations into gene structure, protein motif composition, gene duplication events, collinearity relationships, and *cis*-acting regulatory elements. Furthermore, the expression patterns of distinct *TaAMT* members were examined under varying ammonium supply conditions and pathogen stress. **Results**: In this study, a total of 21 *TaAMT* members were identified. Additionally, all TaAMT proteins were localized to the plasma membrane. Phylogenetic analysis clustered these genes into four distinct subgroups. Comparative analyses of gene structure and conserved motifs revealed conserved domain composition and motif organization within each subgroup. Interspecific synteny analysis highlighted evolutionary conservation across species. Promoter region analysis identified multiple *cis*-regulatory elements associated with hormone signaling, light responsiveness, and abiotic stress adaptation. Expression profiling demonstrated that *TaAMT* members exhibit both tissue-specific and constitutive expression patterns across developmental stages. RT-qPCR further revealed that the expression of *TaAMT* members responds to varying concentrations of ammonium nitrogen supply, as well as infection stresses caused by stripe rust and powdery mildew. **Conclusions**: Collectively, this study uncovered the functional diversity of *TaAMT* members, offering novel molecular targets and theoretical foundations for breeding wheat varieties with enhanced nitrogen use efficiency and disease resistance.

## 1. Introduction

Nitrogen (N) is an indispensable macronutrient for plant growth and development, playing a pivotal role in various physiological processes [1,2]. Among the diverse forms of nitrogen available in the soil, ammonium (NH_4_^+^) serves as a primary nitrogen source for many plants, especially in environments where nitrification is inhibited, such as acidic soils or waterlogged conditions [3]. The efficient acquisition and assimilation of ammonium in plants predominantly rely on ammonium transporters (AMTs), which facilitate the selective movement of NH_4_^+^ across cellular membranes [4,5]. Given their critical role in nitrogen acquisition and homeostasis, *AMT* genes have garnered significant attention in plant physiology and molecular biology research.

To date, genome-wide identification and characterization of the *AMT* gene family have been conducted in several plant species [6,7,8,9], such as *Arabidopsis thaliana*, rice (*Oryza sativa*) [10], and maize (*Zea mays*) [11,12], revealing the diversity, structural features, and functional roles of these genes. In *Arabidopsis thaliana*, six *AtAMTs* were identified and divided into two subfamilies (*AMT1* and *AMT2*), of which five belong to the *AMT1* subfamily (*AtAMT1.1*–*AMT1.5*), and only one (*AtAMT2*) belongs to the *AMT2* subfamily. AMT1 proteins are primarily involved in high-affinity NH_4_^+^ transport, while AMT2 members play a role in low-affinity transport and metabolic regulation [13,14]. The different AMT subtypes exhibit variations in ammonium ion substrate affinity and transport activity [15]. The *AMT1* subfamily plays a dominant role in high-affinity NH_4_^+^ absorption [16]. For example, *AtAMT1.1* and *AtAMT1.3* respond to changes in NH_4_^+^ concentration via phosphorylation, forming a dynamic regulatory loop characterized by low-nitrogen-induced activation and high-nitrogen-suppressed degradation [13,17,18].

Wheat (*Triticum aestivum* L.) is one of the most important staple crops globally, providing a major source of calories and protein for human consumption. Nitrogen (N) nutrition is a critical determinant of crop productivity and grain quality. The first “Green Revolution” in the 1960s [19,20], marked by the breeding of new dwarf wheat varieties, led to a dramatic increase in global wheat production and resolved the food crisis caused by explosive population growth worldwide [21,22,23]. However, the low nitrogen use efficiency (NUE) of dwarf varieties forces high fertilizer use, which increases cultivation costs and causes environmental pollution [24,25]. Thus, breaking the breeding bottleneck to achieve high yield with high nitrogen efficiency is crucial for sustainable agriculture [26,27]. Notably, compared to ammonium-preferred crops like rice, wheat exhibits significantly lower affinity for ammonium nitrogen (NH_4_^+^), and the activity of its key nitrogen assimilation enzyme (e.g., glutamine synthetase, GS) is highly susceptible to environmental fluctuations [28,29]. These characteristics make wheat’s NUE even more complex to improve [30]. Thus, systematically dissecting the molecular regulatory mechanisms underlying wheat nitrogen absorption and breaking through its “high-input, low-efficiency” utilization bottleneck have become core scientific issues for achieving the agricultural goal of “reduced nitrogen input with enhanced efficiency” [27,31]. Furthermore, previous studies have shown that *AMT1* can positively influence sheath blight (*ShB*) resistance by regulating ammonium uptake and assimilation [32], and studies have also indicated that ammonium transporter (AMT) genes are also involved in wheat’s defense responses against stem rust [33] and stripe rust [34]. This raises an interesting question of whether *TaAMT* gene family members may play a role in other pathogen defense responses, and their expression dynamics under NH_4_^+^ stress and pathogen challenge remain poorly understood.

This study performed a genome-wide identification of the *AMT* gene family in wheat and investigated their phylogenetic relationships, gene structures, and expression profiles under ammonium nutrition and pathogen infection. The results may contribute to a deeper understanding of the biological functions of *TaAMT* members, and provide novel insights into the dual roles of *TaAMT* members in N nutrition and biotic stress adaptation, offering potential targets for breeding wheat varieties with improved NUE and disease resistance [35].

## 2. Materials and Methods

### 2.1. Experimental Materials and Treatment

The experimental materials included the wheat varieties Kenong 199 (KN199), 7182, and the wheat-*Thinopyrum ponticum* disomic alien substitution line CH10A5 [36]. The seeds were disinfected with 1% sodium hypochlorite for 10 min, rinsed with distilled water, and then germinated at 25 °C in the dark for 24 h. Uniformly germinated seedlings (with radicles 1–2 cm long) were selected and transferred to hydroponic boxes (containing 2 L of wheat nutrient solution, pH = 5.5, Coolaber NSP1070). They were then cultivated in a light incubator (16 h light/8 h dark, light intensity 300 μmol·m^−2^·s^−1^, temperature 22 ± 2 °C).

For ammonium salt stress treatment, three-leaf seedlings were exposed to nutrient solutions with 0.04 mM (low nitrogen), 0.4 mM (normal nitrogen, CK), and 40 mM (high nitrogen) NH_4_^+^. Samples (0.5 g of the second top leaf) were collected at specified times (0, 6, 12, 24, 36, 48, 72, 96, 144, 168, and 216 h), frozen in liquid nitrogen, and stored at −80 °C, with three biological replicates per treatment.

For pathogen inoculation, seedlings at the two-leaf and one-heart stage were inoculated with stripe rust race CYR34 and powdery mildew race E09, respectively. Samples were taken at 0, 24, and 48 h post-inoculation, frozen in liquid nitrogen, and stored at −80 °C, with three biological replicates per treatment.

### 2.2. Identification and Physicochemical Property Analysis of the AMT Gene Family in Wheat

Reference genomes, protein sequences, and GFF3 annotations for *Arabidopsis thaliana* (TAIR 10), rice (MSU 7.0), and wheat (RefSeq v1.1) were downloaded from Ensembl Plants [37]. *Arabidopsis thaliana* AMT proteins (https://www.arabidopsis.org/) were used as queries in local BLAST 2.4.0+ (E-value < 1 × 10^−5^) against the wheat proteome to identify candidate TaAMT sequences. The *AMT* family HMM profile (PF00909) from Pfam (http://pfam.xfam.org/) was searched in wheat proteins using HMMER 3.3 (HMMsearch, E-value < 1 × 10^−10^). Candidates were validated for conserved AMT domains (PF00909) via CDD (https://www.ncbi.nlm.nih.gov/cdd, accessed on 17 December 2024) and SMART (http://smart.embl-heidelberg.de/, accessed on 17 December 2024). Physicochemical properties (Mw, pI, instability index, aliphatic index) of TaAMT proteins were predicted using ExPASy ProtParam (https://web.expasy.org/protparam/, accessed on 18 December 2024). *TaAMT* members were renamed based on chromosomal positions.

### 2.3. Phylogenetic Analysis of TaAMT Proteins

AMT protein sequences from Arabidopsis, rice, and wheat were aligned using Clustal X 2.1 (default parameters: Gap Opening Penalty = 15, Gap Extension = 6.66, DNA Weight Matrix = IUB) [38]. A phylogenetic tree was constructed with MEGA 11.0 via the Maximum Likelihood (ML) method, using the JTT+G+I substitution model selected by Model Test (1000 bootstrap replicates) [39]. The tree was visualized and annotated with species origins and gene names using iTOL (https://itol.embl.de/upload.cgi, accessed on 11 March 2025) [40].

### 2.4. Analysis of Gene Structure, Protein Features, and Cis-Regulatory Elements

*TaAMT* member structures were analyzed using TBtools v2.225 to extract exon/intron counts, lengths, and positions from wheat GFF3 annotations and generate schematic diagrams. Conserved motifs in TaAMT proteins were identified with MEME Suite v5.0.5 (motif width: 6–50 aa; max motifs: 15; default EM algorithm settings, 100 iterations) [41]. Promoter sequences (2000 bp upstream) were retrieved from WheatOmics 1.0 [42] (http://wheatomics.sdau.edu.cn/, accessed on 2 January 2025) and analyzed for *cis*-elements using PlantCARE (https://bioinformatics.psb.ugent.be/webtools/plantcare/html/, accessed on 2 January 2025). The results were visualized with TBtools v2.225 [43].

### 2.5. Chromosomal Distribution, Gene Duplication Events, and Synteny Analysis of TaAMTs

The chromosomal physical positions of *TaAMT* members were extracted from the wheat GFF3 annotation file, and their distribution maps on chromosomes were generated using TBtools v2.225 [44]. Gene duplication events (tandem and segmental) within the *TaAMT* family were identified via MCScanX (http://chibba.pgml.uga.edu/mcscan2/#tm, accessed on 24 January 2025) using wheat RefSeq v1.1 synteny data (E-value < 1 × 10^−10^). The synteny analysis results were visualized using the built-in module of MCScanX.

### 2.6. Analysis of Tissue Expression Patterns of TaAMTs

Transcriptome data of *TaAMTs* genes across stages and tissues were downloaded from ExpVIP (http://www.wheat-expression.com/, accessed on 17 March 2025). The tissues and developmental stages selected for the expression analysis included roots (seven stages), leaves (six stages), stems (five stages), spikes (one stage), and grains (five stages), totaling 132 samples. The expression data of *TaAMT* members were standardized using Z-score normalization with TBtools v2.225, and hierarchical clustering heatmaps were generated with log_2_(FPKM+1) as the metric. Colors in heatmaps indicate expression levels (red: high; blue: low), with Euclidean distance used for clustering.

### 2.7. Analysis of Expression Patterns of TaAMTs Under Different Stress Conditions

According to the ExpVIP database, a total of ten representative *TaAMT* members with tissue-specific expression were selected to analyze their expression dynamics under ammonium stress, stripe rust (CYR34), and powdery mildew (E09) infection via RT-qPCR. Additionally, the *TaActin* served as the reference gene, with amplification on the QuantStudio TM^6^ Flex system using TBGreen^®^ Premix ExTaq^TM^ II (Tli RNaseH Plus, RR820A, TaKaRa). Three biological replicates were set per time point. Relative expression was calculated by the 2^−ΔΔCT^ method, and significant differences among treatments were determined using one-way ANOVA and Duncan’s test (*p* < 0.01) in SPSS 26.0. The primers used in this study were designed using Primer Premier 5 (version 5.0) and synthesized by Shanghai Sangon Biological Engineering Technology Co., Ltd, Shanghai, China. The primer sequences were listed in Table A1.

## 3. Results

### 3.1. Identification and Basic Physicochemical Properties of TaAMT Members

Based on the Chinese Spring reference genome data, a total of 21 *TaAMT* family members were identified and named according to their chromosomal locations (Supplementary Table S1). Physicochemical property analysis showed that TaAMT proteins range in length from 466 to 563 amino acids, with relative molecular masses of 49,910.41 to 59,889.76 Da and theoretical isoelectric points (pI) ranging from 6.30 to 9.13, averaging 7.52. The total grand average of hydrophobicity (GRAVY) ranged from 0.420 to 0.630, indicating that all members are hydrophobic (Table 1). All TaAMT members were localized on the plasma membrane, indicating that these genes play regulatory roles in the plasma membrane.

### 3.2. Phylogenetic Tree Analysis, Conserved Domains, and Gene Structural Features

**Figure 1:**
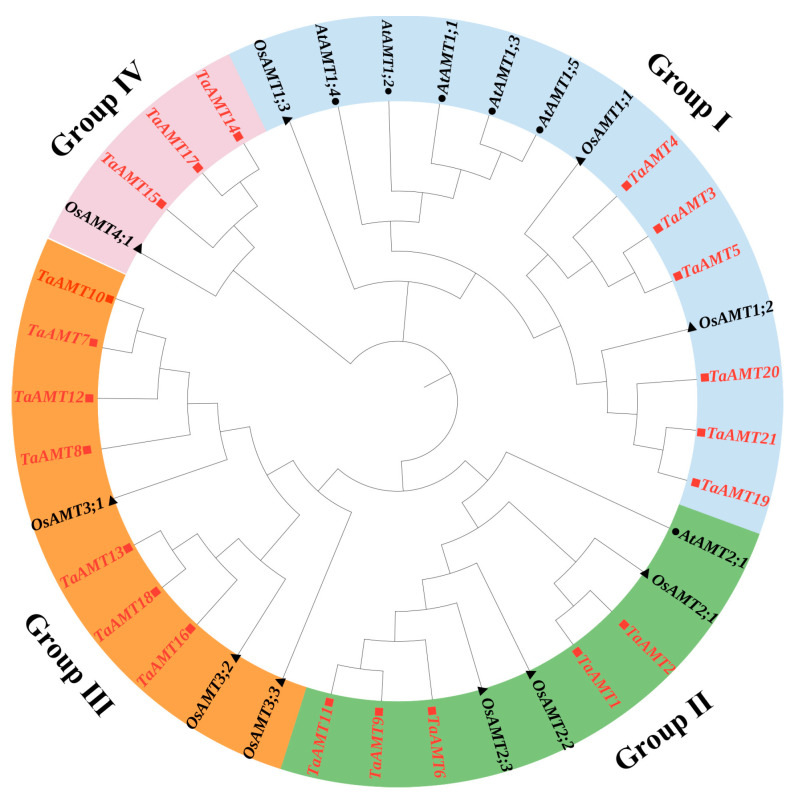
Phylogenetic analysis of AMT proteins, derived from *Arabidopsis thaliana* (At, circle, in black), *Oryza sativa Japonica* (Os, triangle, in black), and *Triticum aestivum* (Ta, square, in red). The tree was constructed using the maximum likelihood (ML) method implemented in MEGA 11 (version 11.0.13).

**Figure 2:**
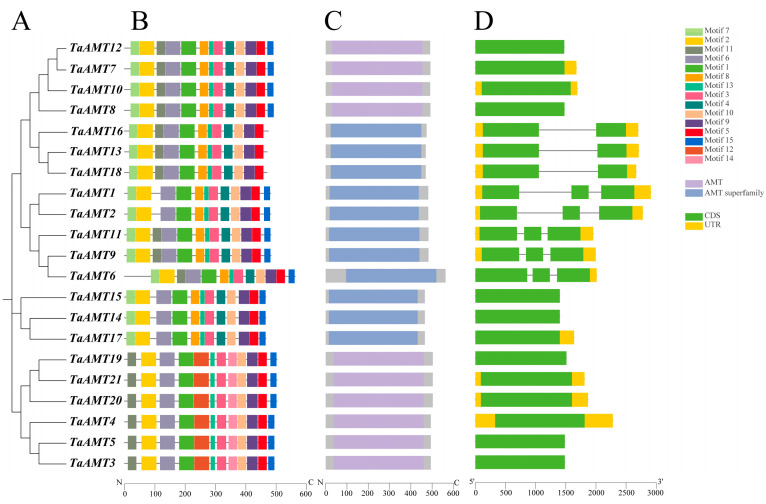
Comprehensive analysis of *TaAMT* gene family members: (**A**) Phylogenetic tree showing evolutionary relationships. (**B**) MEME-based visualization of conserved motifs, with colored boxes representing specific conserved amino acid sequences. (**C**) Conserved domain architecture inferred from NCBI-CDD analysis, with color-coded boxes indicating phylogenetically conserved functional modules. (**D**) Gene structure analysis depicting exon–intron organization of *TaAMT* members.

A phylogenetic tree was constructed using homologous proteins from *Arabidopsis thaliana*, rice, and wheat, which categorized the *TaAMT* family members into four distinct groups (Figure 1). Notably, Groups I and II clustered with *AtAMT1*, whereas Groups III and IV aligned with *AtAMT2*, mirroring the classification pattern observed in *OsAMTs*. We identified 15 conserved motifs within the TaAMT proteins via MEME Suite 5.0.5, with proteins belonging to the same group or subgroup displaying similar motif compositions (Figure 2A,B). Specifically, motifs 1, 2, 3, 6, 9, 10, 12, and 13 emerged as the core motifs conserved across the family. Group, encompassing six members, exhibits high homology to *OsAMT1*, encompassing 11 motifs, including the distinctive motifs 12 and 14. Conversely, Groups II (five members), III (five members), and IV (three members) show homology to *OsAMT2*, *OsAMT3*, and *OsAMT4*, respectively. Gene structure analysis revealed that members of Groups I and IV harbor a single exon, Group IV contains three exons, and Group III possesses either one or two exons (Figure 2D). These findings suggest that these structural discrepancies may potentially arise from gene duplication events, subsequently leading to functional divergence.

### 3.3. Chromosomal Localization, Synteny, and Interspecific Evolution

**Figure 3:**
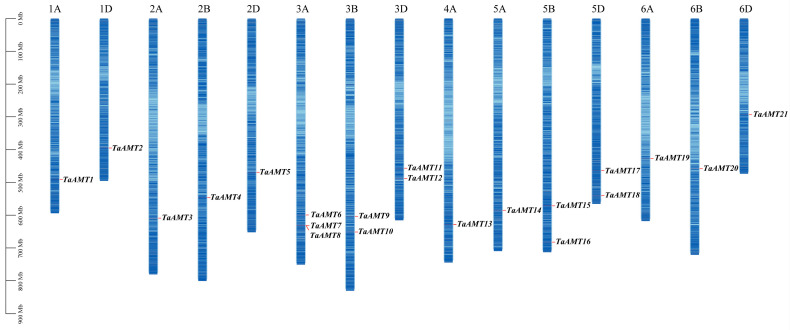
Chromosome distribution of *TaAMT* members in the wheat genome.

**Figure 4:**
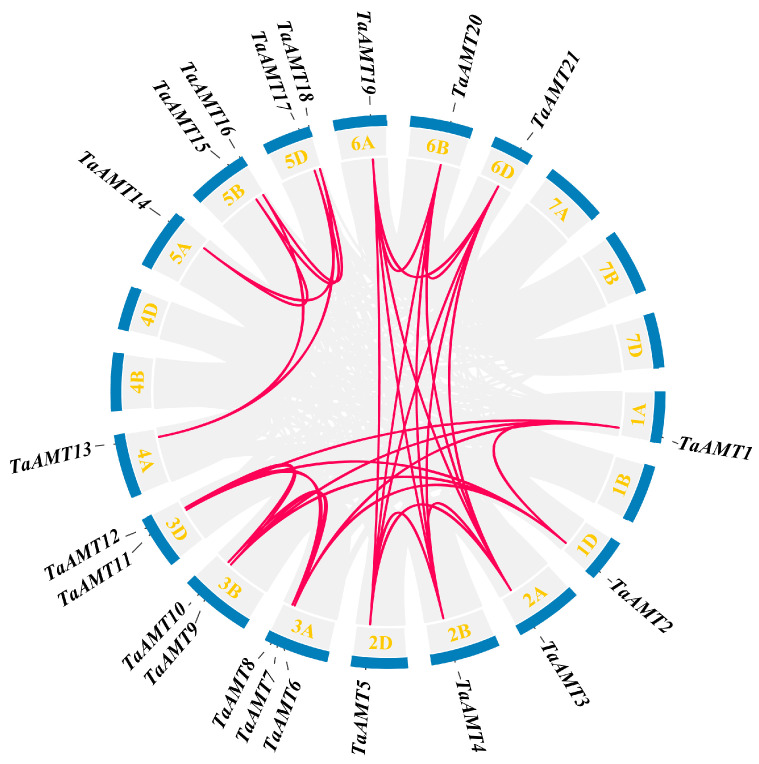
Genomic collinearity analysis of *TaAMT* gene family members. Red lines indicate gene pairs with collinearity relationships; numbers within the boxes represent chromosomes, and light blue boxes denote gene density. This figure was generated using TBtools v2.225.

**Figure 5:**
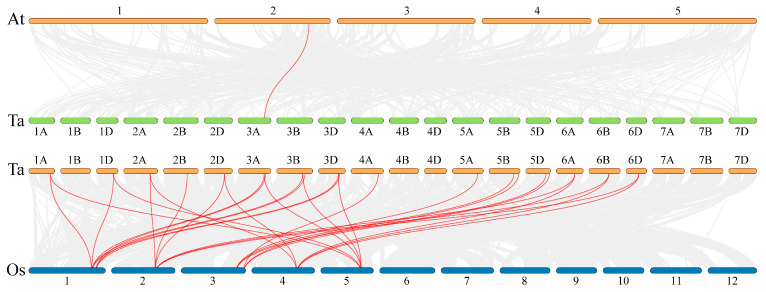
Interspecific collinearity analysis of *AMT* gene family members. Red lines indicate collinear gene pairs between wheat (Ta) and other plant species, with chromosomal positions shown in circular layout. Species abbreviations: Ta, *Triticum aestivum*; At, *Arabidopsis thaliana*; Os, *Oryza sativa*. This diagram was generated with TBtools software (version 2.225).

Chromosomal localization analysis revealed an uneven distribution of 21 *TaAMT* gene family members across 15 wheat chromosomes, all located on the long arms (Figure 3). Notably, the first homology group harbored merely 2 genes (9.5%), whereas the third homology group exhibited the highest gene count, with 7 genes (33.3%). Synteny analysis identified 34 gene pairs within the wheat genome (Figure 4). Among these, paralogous gene pairs situated on homologous chromosomes predominated. Interspecific analysis reveals 1 gene pair between *Arabidopsis thaliana* and wheat (3A), and 30 between wheat and rice, with wheat chromosome 3 and rice chromosome 1 showing high linear consistency in *AMT* gene evolution, providing clues for cross-species origin studies. Inter-species synteny analysis (Figure 5) showed evolutionary conservation, with only one syntenic pair between *Arabidopsis thaliana* and wheat, but 30 between wheat and rice, especially a high degree of linear consistency between wheat chromosome three and rice chromosome one in *AMT* gene evolution, which provides key clues for studying the cross-species origin of the *AMT* family.

### 3.4. Cis-Acting Element Analysis

**Figure 6:**
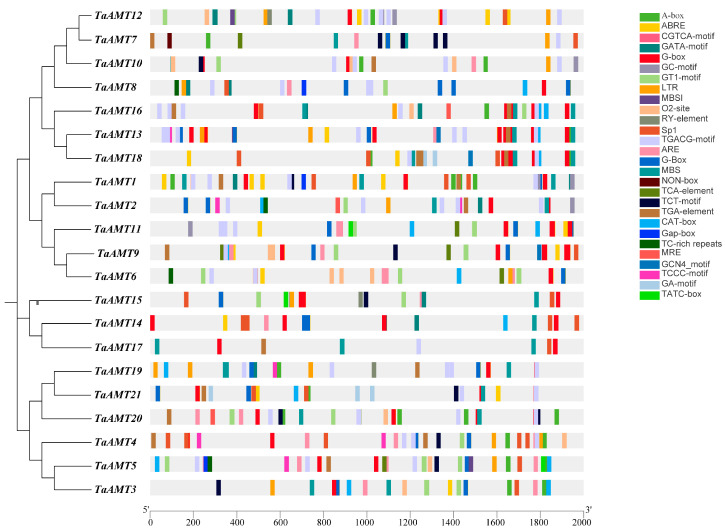
Analysis of cis-acting elements in *TaAM*T members. Boxes of different colors represent distinct cis-acting elements, with their names listed on the right. This figure was generated with TBtools (v2.225).

The prediction of *cis*-acting elements extraction from *TaAMT* members revealed that all *TaAMT* gene family members collectively encompass a rich diversity of elements (Figure 6). These elements are associated with hormone responses and abiotic stresses, with hormone-responsive elements being the most prevalent, covering 10 distinct types. Among them, methyl jasmonate (MeJA)-related elements (CGTCA motif and TGACG motif) were particularly abundant, totaling 124, closely followed by abscisic acid (ABA)-responsive elements (ABREs) with 114 occurrences, while elements responsive to auxin, gibberellin, and salicylic acid were present in smaller numbers. Notably, all *TaAMT* members contain the ABA-responsive element ABRE, suggesting their universal involvement in ABA-mediated stress responses or developmental regulation. Additionally, five types of abiotic stress-related elements were identified, including drought-responsive MBS, low-temperature-responsive LTR, hypoxia-responsive ARE, and GC-motif elements, along with a limited number of metal ion stress-related MRE, general stress-responsive MBSI, and NON-box elements. The presence of these elements strongly implies that the *TaAMT* family genes may dynamically regulate ammonium nitrogen uptake in response to environmental cues such as drought, low temperature, and hypoxia, thereby enhancing the plants’ ability to adapt and thrive under adverse conditions.

### 3.5. Tissue-Specific Expression Pattern Analysis

**Figure 7:**
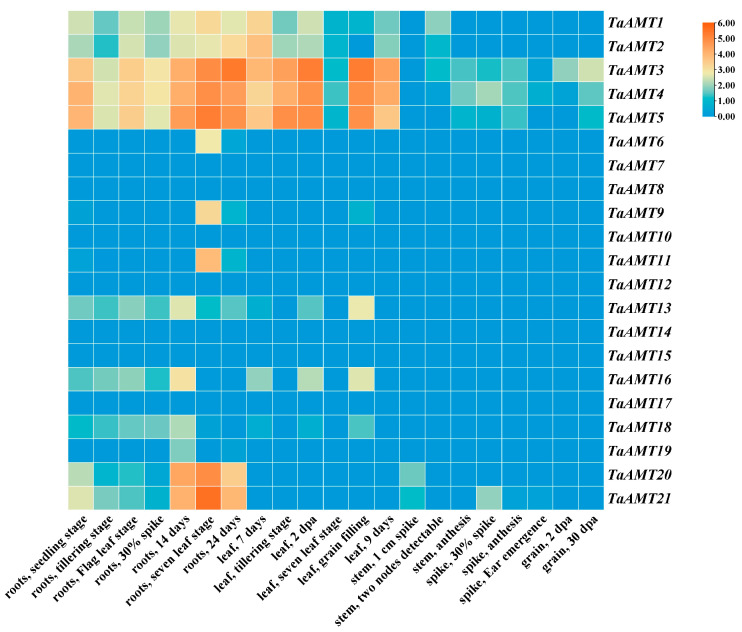
Heatmap of *TaAMT* members’ expression across different growth stages from the ExpVIP database.

Based on the transcriptome data from the ExpVIP database, a heatmap depicting the expression patterns of *TaAMT* members was generated (Figure 7). The results revealed that the 21 *TaAMT* members exhibited notable tissue-specific expression patterns, which could be categorized into three groups: Firstly, there were three constitutively expressed genes showing expression across all examined tissues, likely participating in the fundamental nitrogen metabolism processes in wheat. Secondly, seven genes displayed constitutively low expression, meaning their expression levels were extremely low in all tissues, and their functions require further verification. Additionally, 11 genes exhibited tissue-specific expression, with significant variations in expression levels across different tissues. Among them, *TaAMT1*, *TaAMT2*, *TaAMT6*, *TaAMT9*, *TaAMT11*, *TaAMT20*, and *TaAMT21* were highly expressed in roots, suggesting their potential involvement in ammonium nitrogen uptake by roots. *TaAMT1*, *TaAMT2*, *TaAMT13*, and *TaAMT16* showed high expression in leaves, possibly related to nitrogen transport or assimilation in the above-ground parts. Notably, *TaAMT21* had significantly higher expression levels in stems and spikes compared to other tissues, indicating its crucial role in nitrogen allocation during the reproductive growth stage. The diversity of these tissue-specific expression patterns provides a molecular basis for the coordinated regulation of nitrogen utilization in wheat by the *TaAMT* gene family members at different developmental stages.

### 3.6. Analysis of Expression Patterns of TaAMT Members Under Three Stress Conditions

**Figure 8:**
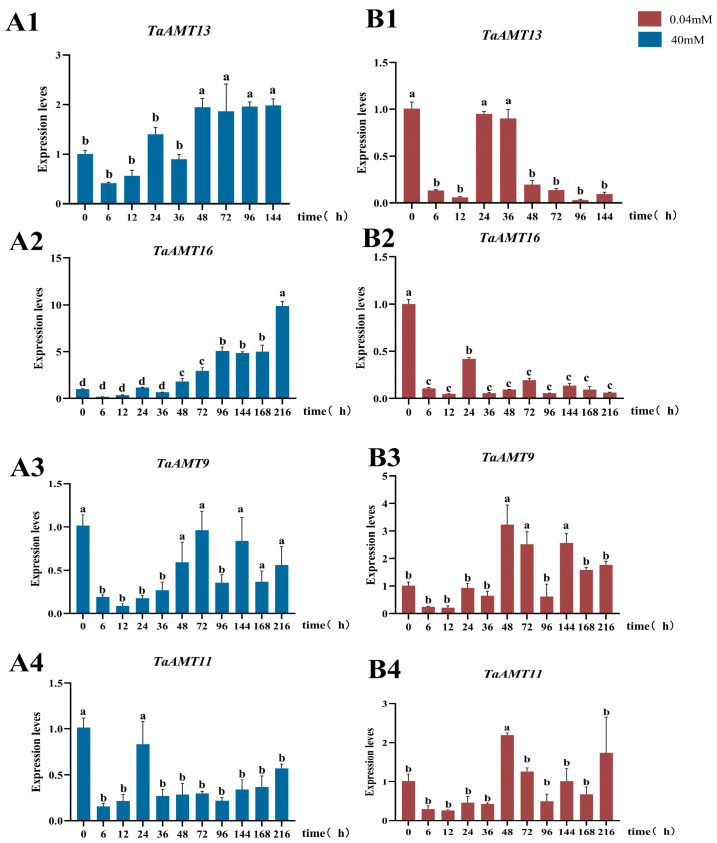
Analysis of expression patterns of four *TaAMT* members under high-ammonium ((**A1**–**A4**), blue, 40 mM) and low-ammonium ((**B1**–**B4**), red, 0.04 mM) stress conditions. The numbers 1 to 4 represent the genes *TaAMT13*, *TaAMT16*, *TaAMT9*, and *TaAMT11*, respectively. Values shown are the means ± SD of three replicates. For each datum, bars with different letters indicate significant differences (Duncan’s test, at *p* < 0.01).

According to the heatmap of *TaAMT* members’ expression across different growth stages from the ExpVIP database, two genes *(TaAMT13*, *TaAMT16*) that were highly expressed only in the roots and two genes (*TaAMT9*, *TaAMT11*) that were expressed in both the roots and leaves were used to further explore *TaAMT* members’ responses to ammonium nitrogen stress. Additionally, their expression was analyzed under high (40 mmol/L NH_4_^+^) and low (0.04 mmol/L NH_4_^+^) ammonium stresses using RT-qPCR. The results showed that *TaAMT13* and *TaAMT16* exhibited delayed upregulation under low ammonium at 72 h, suggesting a role in low-nitrogen absorption. Under high ammonium, their expression reversed, downregulated to 45% and 52% of control at 48 h, possibly avoiding cytotoxicity (Figure 8). Conversely, *TaAMT9* and *TaAMT11* were downregulated at 12 h under low ammonium, but rebounded to 1.6–1.8 times control at 48 h under high ammonium, indicating dynamic ammonium sensing and nitrogen homeostasis regulation.

**Figure 9:**
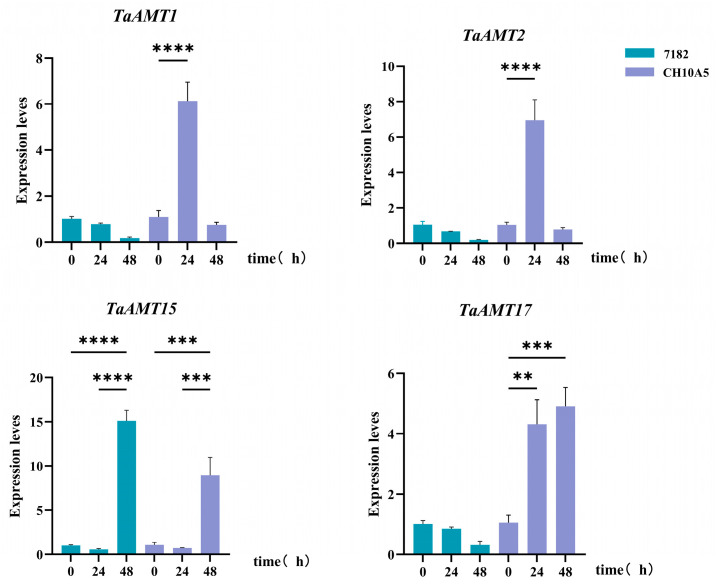
Expression patterns of four *TaAMT* members under stripe rust stress (CYR34). **, ***, and **** indicate the significant correlations at the levels of *p* < 0.01, *p* < 0.001, and *p* < 0.0001, respectively.

Utilizing the ExpVIP database, four candidate genes potentially involved in stripe rust resistance were selected. Additionally, their expression dynamics were subsequently analyzed in the wheat varieties 7182 and CH10A5 following inoculation with CYR34. At 24 h post-inoculation, *TaAMT1*, *TaAMT2*, and *TaAMT17* were significantly upregulated, likely linked to defensive nitrogen metabolism. At 48 h, *TaAMT15* expression peaked at 2.1–2.4 times control, coinciding with hyphal expansion, suggesting its role in disease resistance nitrogen signaling (Figure 9).

**Figure 10:**
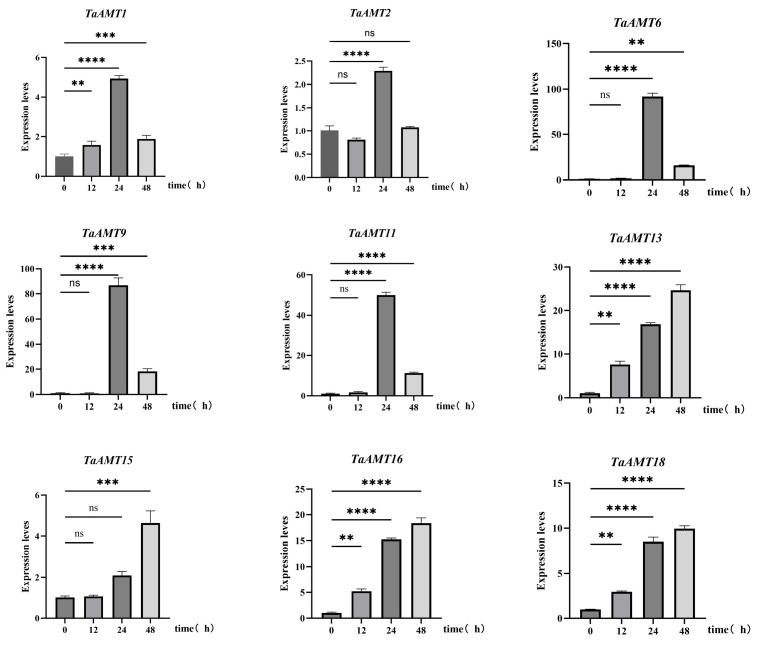
Gene expression patterns of nine *TaAMT* members under powdery mildew stress (E09). **, ***, and **** indicate the significant correlations at the levels of *p* < 0.01, *p* < 0.001, and *p* < 0.0001, respectively; ns indicate no significant difference.

Under powdery mildew stress induced by E09 infection, nine members of the *TaAMT* gene family exhibited time-course-specific expression patterns. Specifically, *TaAMT1* peaked at 24 h (5-fold), while *TaAMT2* was transiently induced at 24 h. *TaAMT6* surged 13-fold at 24 h. *TaAMT9* and *TaAMT13* had transient peaks at 24 h (10–15-fold). *TaAMT11* mirrored *TaAMT9* but with a weaker peak. *TaAMT15* was upregulated only at 24 h, *TaAMT16* remained high at 24 h and 48 h, and *TaAMT18* peaked at 48 h (Figure 10).

Overall, *TaAMT* gene family members show gene-specific, time-dependent expression under ammonium stress and coordinated responses to stripe rust and powdery mildew infections, suggesting their key roles in ammonium homeostasis regulation and disease resistance via pathogen signal sensing, offering vital candidates for exploring “nitrogen–disease” interaction mechanisms.

## 4. Discussion

Nitrogen is an essential component of important organic compounds within plants [45]. Different wheat germplasm resources [46] exhibit varying nitrogen fertilizer uptake capacities under different environmental factors [27,47,48]. Key components of signal transduction pathways, such as ubiquitin ligases and transcription factors, play a central role in the processes by which plants perceive and respond to nutrient changes [49,50,51,52]. Epigenetic modifications also participate in regulating the efficiency of nitrogen transport and utilization in plants [53]. This study conducted a systematic examination of the evolutionary traits, expression patterns, and stress response mechanisms within the wheat *AMT* gene family members, highlighting its pivotal role in nitrogen homeostasis and disease resistance. The findings bridge gaps in *TaAMT* gene family members’ functional research and lay a theoretical foundation for molecularly designing wheat varieties with “reduced nitrogen input, enhanced efficiency.”

### 4.1. Evolutionary Dynamics and Functional Divergence of TaAMT Gene Family Members

The evolutionary trajectory of the *TaAMT* gene family members was closely tied to wheat polyploidization. Phylogenetic analysis categorizes *TaAMT* into four clusters, aligning with *Arabidopsis thaliana* and rice classifications, suggesting conserved functional divergence in monocots. Compared to *Arabidopsis thaliana* (6) and rice (8), wheat boasts a larger repertoire with 21 *AMT* members, likely due to polyploidy-driven gene expansion. Chromosomal mapping reveals a high concentration on homologous Group III, with collinearity analysis indicating segmental and tandem duplications as key expansion mechanisms [54,55]. Motif and gene structure analyses underscore functional differentiation, with Group I members potentially mediating high-affinity transport, while Group III members may be involved in low-affinity or adaptive regulation. All *TaAMT* members localize to the plasma membrane, reinforcing their role as ammonium transporters [56,57]. The synteny analysis strongly suggests that the expansion of the *TaAMT* family primarily hinges on gene duplication events occurring between homologous chromosomes and may have undergone truncated duplication during the course of evolution.

### 4.2. Cis-Regulatory Elements and Stress Response Mechanisms

Analysis of *cis*-acting elements in the promoter regions revealed a regulatory network governing *TaAMT* members’ responses to diverse signals. All *TaAMT* gene family members harbor ABA-responsive elements (ABREs). Given their expression patterns under abiotic stresses such as drought and low temperature [58], it is hypothesized that ABA may serve as a key signaling molecule for *TaAMT* members in response to environmental stresses. Specifically, ABA activates the transcription of *TaAMT* members by binding to ABREs, thereby promoting ammonium uptake to maintain nitrogen homeostasis under stress conditions [59]. *NRT1.1B* has been confirmed to function as an ABA receptor [60], and when plants utilize nitrate (NO_3_^−^) as a nitrogen source, ABA levels decrease compared to those when ammonium (NH_4_^+^) is used [61,62]. Consequently, there exists an interplay and mutual influence between the regulatory networks of AMTs and ABA. Additionally, MeJA-related elements are the most abundant (124 elements), suggesting that jasmonic acid signaling may be involved in the defensive regulation of *TaAMT* members. Previous studies have demonstrated that jasmonic acid can enhance plant disease resistance by inducing the expression of defense-related genes [63,64], and the upregulation of *TaAMT* members (e.g., *TaAMT1* and *TaAMT2*) during the early stages of stripe rust infection may act synergistically with this pathway.

### 4.3. Tissue-Specific Expression and Dynamic Regulation of Nitrogen Uptake and Utilization

The tissue expression heatmap from the ExpVIP database revealed a distinct tissue-specific division of labor among *TaAMT* members, namely *TaAMT1*, *TaAMT2*, *TaAMT6*, and others exhibited high expression in roots, suggesting their dominant role in root ammonium uptake. *TaAMT13* and *TaAMT16* showed high expression in leaves, potentially participating in nitrogen translocation or assimilation in the shoot. *TaAMT21* was specifically highly expressed in stems and spikes, implying its regulatory role in nitrogen allocation during reproductive growth stages. This tissue specificity is highly conserved compared to the patterns of *AtAMT1*;1 and *OsAMT1*;1 [15,65], reflecting the central role of *AMT* genes in plant nitrogen uptake.

Notably, in contrast to their constitutive high expression in roots, *TaAM*T9 and *TaAMT11* were rapidly suppressed by low ammonium within 12 h but strongly induced by high ammonium at 48 h. This dynamic pattern of “low ammonium inhibition-high ammonium activation” resembles the phosphorylation-mediated regulation mechanism of *AtAMT1;3*. It likely involves perceiving extracellular NH_4_^+^ concentration changes to dynamically adjust transport activity, thereby avoiding energy waste under low nitrogen conditions or toxicity accumulation under high nitrogen conditions [18]. In contrast, *TaAMT13* and *TaAMT16* exhibited a compensatory pattern of “delayed upregulation under low ammonium-rapid downregulation under high ammonium”. This implies that they may synergistically maintain nitrogen homeostasis by delaying the activation of ammonium uptake pathways under low ammonium concentrations and implementing negative feedback inhibition under high ammonium concentrations. Research has shown that early low nitrogen pretreatment can enhance nitrogen uptake and assimilation capacity in wheat seedlings, ensuring normal crop growth and high yield [66]. Under appropriate high ammonium conditions, plants can utilize complex regulatory systems to ensure adequate nitrogen uptake while avoiding ammonium toxicity [67,68].

### 4.4. Pathogen Infection Responses and the “Nitrogen–Disease” Nexus

The expression of *TaAMT1*, *TaAMT2*, and *TaAMT17* was significantly upregulated 24 h after infection with the stripe rust fungus, indicating their potential involvement in nitrogen metabolism reprogramming during early defense responses [69,70]. Pathogen invasion induces nitrogen uptake in the host to support the synthesis of defensive compounds [71]. Meanwhile, *TaAMT15* was upregulated after 48 h, with its peak expression coinciding with a critical phase of hyphal expansion (48–72 h), suggesting its role in activating downstream defense genes through nitrogen signaling. Under stripe rust stress, some genes exhibited marked upregulation, potentially linked to defensive nitrogen metabolism responses and disease resistance-related nitrogen signaling [69].

Following powdery mildew infection, the significant upregulation of *TaAMT1* and TaAMT6 aligns with reports that powdery mildew secretes effector proteins to interfere with host nitrogen uptake, implying that the elevated expression of *TaAMT* may represent a compensatory strategy by the host to counteract pathogen nitrogen exploitation [70]. Additionally, the sustained high expression of *TaAMT16* and *TaAMT18* suggests their involvement in long-term regulation of disease resistance, such as maintaining nitrogen metabolism balance to support continuous synthesis of pathogenesis-related proteins. Notably, distinct temporal patterns and intensities of responses among different *TaAMT* members after powdery mildew infection indicate their potentially diverse functional roles in ammonium homeostasis regulation or defense responses during wheat pathogen infection. This study suggested that *TaAMTs* could influence defense signaling through a cascade of events: primarily by mediating ammonium flux-induced pH changes that alter the cellular signaling milieu; this, in turn, may trigger competition for energy and nitrogen resources between growth and defense processes; and additionally, AMTs might play direct roles in facilitating the production of specific signaling molecules related to ammonium metabolism. However, significant research gaps remain regarding the specific mechanisms of *AMT* genes in powdery mildew, how they perceive pathogen signals, and their involvement in wheat disease resistance. Further investigations are urgently needed to elucidate the molecular mechanisms underlying the “nitrogen–disease” interaction.

## 5. Conclusions

In summary, this study systematically characterized the *TaAMT* gene family members, identifying 21 *TaAMT* members distributed across 15 chromosomes, with distinct physicochemical properties and plasma membrane localization. Phylogenetic analysis grouped *TaAMTs* into four clades, mirroring the evolutionary divergence of *AMT1*/*AMT2* subfamilies in *Arabidopsis thaliana* and rice, suggesting functional specialization in high and low-affinity ammonium uptake. *Cis*-element analysis revealed enrichment of hormone-responsive (e.g., ABA, MeJA) and abiotic stress-related motifs in their promoters, aligning with tissue-specific expression patterns, indicating roles in nitrogen homeostasis under stress. Under ammonium stress, *TaAMT13* and *TaAMT16* showed delayed upregulation under low-N conditions and downregulation under high-N, while *TaAMT9* and *TaAMT11* exhibited dynamic responses, suggesting adaptive nitrogen sensing. During stripe rust and powdery mildew infections, *TaAMT1*, *TaAMT2*, and *TaAMT15* were significantly upregulated, implying dual roles in nitrogen metabolism and disease resistance. These findings provide genetic resources and mechanistic insights for improving nitrogen use efficiency and stress resilience in wheat through targeted breeding.

## Figures and Tables

**Table 1:** Physicochemical parameters of *TaAMT* members.

Name	Number of Amino Acid	Molecular Weight	Theoretical pI	Instability Index	Aliphatic Index	Grand Average of Hydropathicity	Subcellular Localization
*TaAMT1*	482	51273.1	7.75	28.77	102.45	0.541	Plasma Membrane
*TaAMT2*	482	51336.2	8.67	29.15	101.43	0.537	Plasma Membrane
*TaAMT3*	494	52190.17	7.15	19.04	94.11	0.520	Plasma Membrane
*TaAMT4*	494	52298.33	7.15	19.59	93.32	0.523	Plasma Membrane
*TaAMT5*	494	52224.19	7.15	19.04	93.32	0.518	Plasma Membrane
*TaAMT6*	563	59889.76	9.13	38.08	100.36	0.427	Plasma Membrane
*TaAMT7*	492	53474.26	7.63	34.41	96.30	0.420	Plasma Membrane
*TaAMT8*	492	53458.29	7.10	34.55	97.13	0.452	Plasma Membrane
*TaAMT9*	483	51327.01	8.28	31.13	107.52	0.583	Plasma Membrane
*TaAMT10*	491	53372.22	7.63	33.25	96.90	0.434	Plasma Membrane
*TaAMT11*	483	51280.94	8.28	31.53	107.70	0.580	Plasma Membrane
*TaAMT12*	491	53385.21	7.63	34.29	96.90	0.428	Plasma Membrane
*TaAMT13*	470	50042.43	6.49	34.26	103.11	0.625	Plasma Membrane
*TaAMT14*	466	50040.55	7.61	33.81	102.62	0.613	Plasma Membrane
*TaAMT15*	466	49917.42	7.07	32.81	104.72	0.630	Plasma Membrane
*TaAMT16*	474	50497.9	6.30	32.68	102.05	0.596	Plasma Membrane
*TaAMT17*	466	49910.41	7.61	32.72	102.83	0.626	Plasma Membrane
*TaAMT18*	470	50056.46	6.49	34.08	103.11	0.625	Plasma Membrane
*TaAMT19*	503	52851.72	7.62	23.94	91.87	0.514	Plasma Membrane
*TaAMT20*	503	52865.74	7.62	23.94	92.07	0.514	Plasma Membrane
*TaAMT21*	503	52865.1	7.62	24.10	91.67	0.509	Plasma Membrane

Note: Physicochemical properties of proteins were predicted using the online website ExPASy ProtParam (https://web.expasy.org/protparam/, accessed on 30 December 2024).

## Data Availability

The original contributions presented in this study are included in the article. Further inquiries can be directed to the first author.

## Supplementary Materials

The following supporting information can be downloaded at: https://www.mdpi.com/article/10.3390/genes16121451/s1, Table S1: *TaAMT* members renamed.

## Abbreviations

The following abbreviations are used in this manuscript:

AMT	Ammonium transporter
RT-qPCR	Reverse Transcription Quantitative Polymerase Chain Reaction
GRAVY	Grand Average of Hydropathy
pI	isoelectric points
ABA	abscisic acid
MBS	Matrix Binding Site
LTR	Long Terminal Repeat
MRE	Metal Response Element
MBSI	Matrix Binding Site I

## Appendix A

**Table A1:** Primer sequences used in this study.

Gene	Forward Primer Sequence	Reverse Primer Sequence
*TaAMT1*	GGGTGGGACCGAGGCTGAAG	CGTGATGTTGGCGGCGTAGC
*TaAMT2*	CAGTTCGAGTTCGCCGCCATC	TGTAGGAGAGCATGAGCCAGAGC
*TaAMT6*	GGTCCGCCCTCCTCCAGAAG	TGGGTGTGCAGGGTGGTGAG
*TaAMT9*	CCCACGCAGCCCTACTACCC	TGAACGCCATCCACGCCTTG
*TaAMT11*	CTGCTCCTCTCCTACACCGTCTG	ATGACGTAGCCGCCGGAGTAG
*TaAMT13*	CGTCGTCATCCGCTTAGTTGTCC	GTGTCATCGTAGTGCTCGCCATC
*TaAMT15*	CAGTTCGTCTTCGCCGCCATC	CCGACGGTGTAGGAGAAGGTGAG
*TaAMT16*	TCTGTGTTGTCATCCGCCITGTC	GTGTCATCGTAGTGCTCGCCATC
*TaAMT17*	CAGTTCGTCTTCGCCGCCATC	CCGACGGTGTAGGAGAAGGTGAG
*TaAMT18*	GTCGTCATCCGCCTTGTCGTC	GTGTCATCGTAGTGCTCGCCATC
*TaActin*	ACCTTCAGTTGCCCAGCAAT	CAGAGTCGAGCACAATACCAGTTG
