# Supplementary material for: Genome-Wide Identification of the AMT Gene Family in Wheat: Expression Profiles Under Ammonium Nutrition and Pathogen Effects
Source: Genes (Basel). 2025 Dec 4;16(12):1451. doi: 10.3390/genes16121451 (PMC12732959; doi:10.3390/genes16121451)
Supplement: Supplementary file 1 [file genes-16-01451-s001.zip › genes-4022445-supplementary.pdf]

Supplementary Table S1 *TaAMT* members rename

Rename	Gene name
TaAMT1	*TraesCS1A02G295300*
TaAMT2	*TraesCS1D02G296600*
TaAMT3	*TraesCS2A02G365000*
TaAMT4	*TraesCS2B02G383600*
TaAMT5	*TraesCS2D02G362900*
TaAMT6	*TraesCS3A02G350800*
TaAMT7	*TraesCS3A02G381600*
TaAMT8	*TraesCS3A02G381700*
TaAMT9	*TraesCS3B02G383400*
TaAMT10	*TraesCS3B02G414300*
TaAMT11	*TraesCS3D02G344800*
TaAMT12	*TraesCS3D02G374800*
TaAMT13	*TraesCS4A02G352900*
TaAMT14	*TraesCS5A02G388100*
TaAMT15	*TraesCS5B02G393200*
TaAMT16	*TraesCS5B02G520200*
TaAMT17	*TraesCS5D02G398200*
TaAMT18	*TraesCS5D02G519400*
TaAMT19	*TraesCS6A02G226800*
TaAMT20	*TraesCS6B02G254800*
TaAMT21	*TraesCS6D02G208200*
